# Spironolactone therapy in resistant hypertension comorbid with mild obstructive sleep apnea: A retrospective cohort study

**DOI:** 10.1097/MD.0000000000048755

**Published:** 2026-05-15

**Authors:** Chao Fang, Bo Ma, Nuan Xiao, Hongmei Zhao, Simin Ren

**Affiliations:** a Geriatrics Department, Hebei University Affiliated Hospital, Baoding, Hebei, China; b Department of Anesthesiology, Hebei University Affiliated Hospital, Baoding, Hebei, China; c Department of Neurology, Hebei University Affiliated Hospital, Baoding, Hebei, China.

**Keywords:** apnea-hypopnea index, obstructive sleep apnea, resistant hypertension, safety, spironolactone

## Abstract

Mild obstructive sleep apnea (OSA) is often underdiagnosed but contributes to treatment-resistant hypertension through oxygen deprivation and activation of the renin–angiotensin–aldosterone system. Spironolactone, an aldosterone antagonist, may help manage both hypertension and OSA-related symptoms. This study aims to evaluate the effectiveness and safety of spironolactone in patients with mild OSA and resistant hypertension. This retrospective observational cohort study included 96 patients diagnosed with mild OSA and resistant hypertension between December 2022 and October 2023. Patients were grouped according to whether they received spironolactone (20 mg/day) in addition to conventional antihypertensive treatment. Outcomes included blood pressure (office, ambulatory, and daytime), apnea-hypopnea index (AHI), lowest oxygen saturation (SaO_2_), and biochemical parameters (blood urea nitrogen [BUN], potassium, sodium, and creatinine). Missing data were handled using multiple imputation. After 12 weeks, the spironolactone group showed greater reductions in blood pressure compared with the control group: office systolic blood pressure (OSBP) decreased by 5.75 mm Hg (95% CI: −8.84 to −2.66) and office diastolic blood pressure (ODBP) decreased by 3.67 mm Hg (95% CI: −6.46 to −0.88). The AHI improved by 1.85 events/hour (95% CI: −3.23 to −0.47), and SaO_2_ increased by 3.33% (95% CI: 0.93–5.73). No cases of hyperkalemia or clinically significant kidney dysfunction were observed, and the treatment was well tolerated. Spironolactone was associated with improved blood pressure control and modest improvements in sleep-related parameters in patients with mild OSA and resistant hypertension, with a favorable short-term safety profile. The small sample size and relatively young cohort limit the generalizability of the findings.

## 1. Introduction

Obstructive sleep apnea (OSA) is characterized by recurrent upper-airway collapse, apnea, and hypoventilation during sleep, often accompanied by intermittent hypoxemia and sympathetic activation.^[1,2]^ With rising obesity and broader screening, OSA incidence is increasing and affecting younger populations.^[3]^ Although mild OSA often presents atypically, it is closely linked to cardiovascular disease, especially suboptimally controlled hypertension.^[4,5]^ OSA is a major cause of secondary hypertension and is particularly prevalent among patients with resistant hypertension. Mechanistically, OSA can elevate blood pressure via excessive sympathetic drive, renin–angiotensin–aldosterone system (RAAS) activation, endothelial dysfunction, and inflammation. In particular, RAAS dysregulation with elevated aldosterone promotes sodium and water retention, worsening hypertension and potentially aggravating OSA severity. Continuous positive airway pressure (CPAP) remains first-line for OSA and improves respiratory events with modest blood-pressure benefits,^[6]^ yet adherence is often poor in mild OSA, underscoring the need for alternative or adjunctive strategies.^[7]^ Prior studies suggest that spironolactone, a mineralocorticoid receptor antagonist, may reduce upper-airway edema and improve blood pressure control, particularly in resistant hypertension with elevated aldosterone.^[8]^ However, evidence in mild OSA with resistant hypertension is limited. Research question: In adults with mild OSA and treatment-resistant hypertension, does adding spironolactone (20 mg/day) to standard antihypertensive therapy improve blood pressure and sleep-disordered breathing indices versus standard therapy alone? Hypothesis: Spironolactone will yield greater reductions in clinic and ambulatory blood pressure and modest improvements in apnea-hypopnea index (AHI) and nadir oxygen saturation (SaO_2_), without excess adverse events.

Accordingly, we conducted a retrospective observational cohort study to evaluate the effectiveness and safety of adding spironolactone to conventional therapy in this population.

## 2. Objects and methods

### 2.1. Study subjects

This study was a retrospective observational cohort study conducted at Hebei University Affiliated Hospital. We reviewed medical records of patients diagnosed with mild OSA and resistant hypertension between December 2022 and October 2023. Patients were divided into 2 groups based on whether they received spironolactone in addition to standard antihypertensive therapy. The study was approved by the institutional ethics committee (Approval No.: HDFYLL-KY-2024-008), and informed consent was waived due to the retrospective nature of the analysis.

Inclusion criteria: Age 30 to 80 years; Diagnosed with mild OSA by polysomnography (PSG), defined as an AHI between 5 and 15 events per hour; Diagnosed with resistant hypertension, defined as a blood pressure consistently higher than 140/90 mm Hg despite the use of ≥3 antihypertensive medications with different mechanisms (including diuretics) at appropriate doses; Normal renal and liver function, capable of completing follow-up during the trial.

Exclusion criteria: Secondary hypertension (except for primary aldosteronism), such as renal vascular hypertension, Cushing syndrome, pheochromocytoma, etc; Moderate to severe OSA (AHI > 15 events per hour) or currently receiving CPAP therapy; History of severe cardiovascular or cerebrovascular events, such as myocardial infarction, stroke, or hospitalization for heart failure within the past 6 months; Creatinine clearance < 60 mL/min/1.73 m^2^ or significant liver dysfunction; Blood potassium ≥ 5.0 mmol/L or blood sodium < 135 mmol/L; Pregnant or breastfeeding women, individuals with menstrual disorders, or a history of gynecomastia; and Allergic to spironolactone or has used aldosterone receptor antagonists within the past 2 weeks.

Normal liver and kidney function were defined as follows: serum creatinine ≤ 133 μmol/L and eGFR ≥ 60 mL/min/1.73 m^2^; ALT, AST, and total bilirubin levels ≤ 1.5 × ULN; and no history of cirrhosis or active hepatitis.

**Figure 1.**
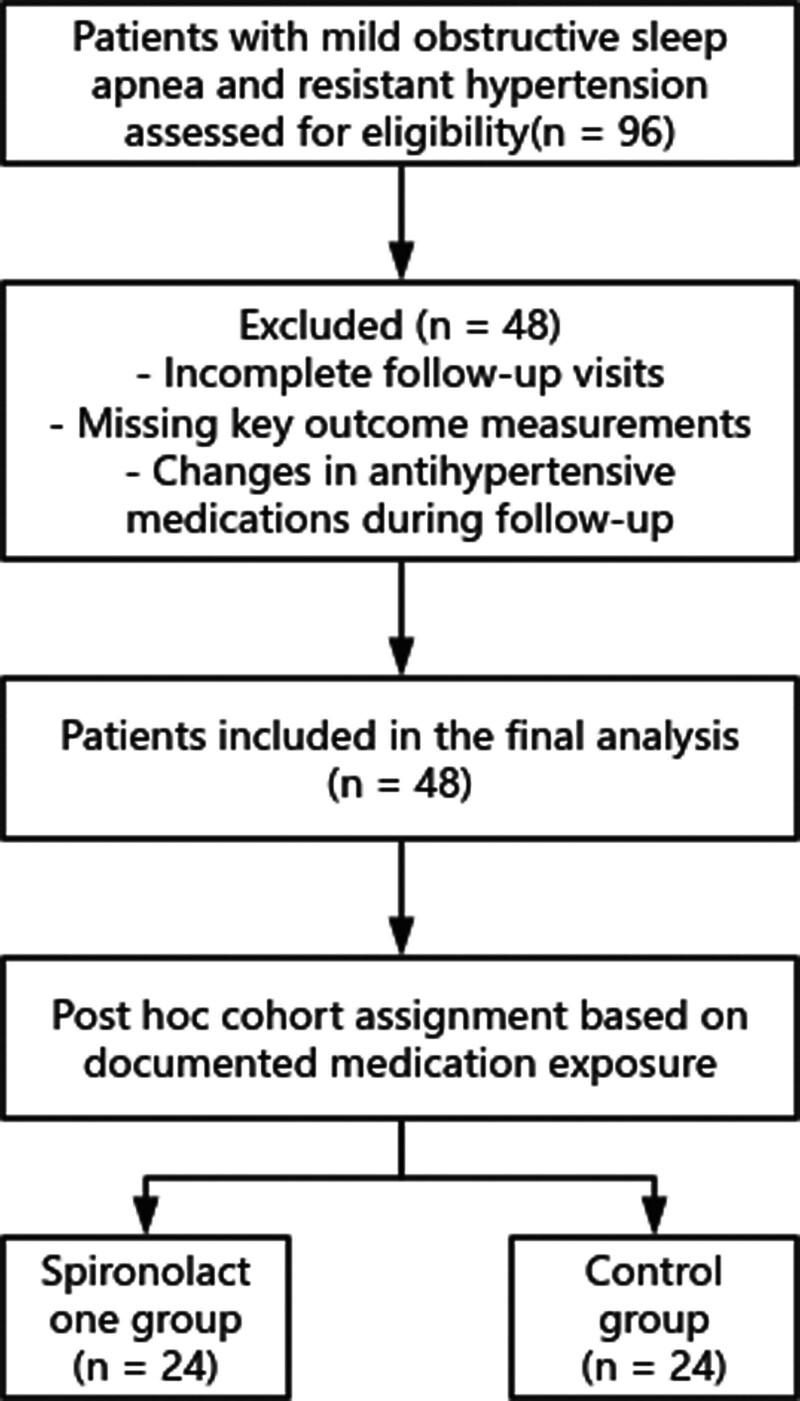
Flowchart of patient selection and cohort assignment.

Patients who met the criteria were assigned to the spironolactone group (n = 24) or control group (n = 24) based on their medication records. Both groups continued their standard antihypertensive regimens throughout the 12-week observation period. Clinical and laboratory data were extracted from hospital records at baseline and at approximately 12 weeks. The process of patient identification, exclusion, and cohort assignment is summarized in Figure 1.

### 2.2. Grouping and intervention methods

Patients who met the inclusion criteria were assigned to cohorts post hoc based on real-world treatment exposure documented in the electronic medical record: those who received spironolactone 20 mg/day in addition to standard antihypertensive therapy formed the spironolactone cohort, and those who continued standard therapy without spironolactone formed the control cohort. Investigators did not control or influence treatment allocation; prescribing decisions were made in routine clinical care.

Both cohorts were managed under otherwise comparable lifestyle and pharmacologic measures, with background antihypertensive regimens kept stable during the 12-week observation. Clinical assessments and laboratory/sleep measurements were extracted at baseline and approximately 12 weeks. Treatment allocation was determined post hoc based on documented medication exposure. Background antihypertensive regimens were stable during the observation period, and no dose adjustments or medication changes were permitted during follow-up.

Of the 96 eligible patients initially identified, 48 completed the 12-week follow-up and were included in the final analysis. The remaining 48 patients were excluded prior to outcome analysis due to incomplete follow-up visits, missing key outcome measurements, or changes in antihypertensive medications during the follow-up period.

#### 2.2.1. Experimental group (spironolactone group)

On the basis of their existing stable antihypertensive regimen, the experimental group received oral spironolactone 20 mg once daily in the morning for a duration of 12 weeks. Spironolactone is an aldosterone receptor antagonist, provided by the hospital pharmacy, with medication adherence education and drug distribution records maintained throughout the study.

#### 2.2.2. Control group

The control group maintained their original antihypertensive regimen without adding spironolactone. Other lifestyle management measures (such as low-salt diet and sleep management) were the same as those in the experimental group, and the observation period was also 12 weeks. Throughout the trial, all participants were required to keep their existing antihypertensive medications and doses unchanged, with no self-adjustment of the medications allowed. The following classes of antihypertensive drugs were permitted, including but not limited to: calcium channel blockers, angiotensin-converting enzyme inhibitors, angiotensin II receptor antagonists (ARB), beta-blockers, and thiazide diuretics.

#### 2.2.3. Monitoring and management during the intervention period

Clinical assessments and laboratory tests were performed for all subjects on day 0 (prior to enrollment), at week 4, and at week 12. Mid-term follow-up was scheduled for week 4 to evaluate blood pressure changes and laboratory indicators, and to monitor for potential adverse reactions. If any of the following occurred – blood potassium ≥ 5.5 mmol/L, significant increase in blood creatinine, severe gastrointestinal reactions, or poor medication adherence – the research team would consider stopping the medication or excluding the patient from the trial analysis.

#### 2.2.4. Adherence and safety assurance measures

All medications were dispensed uniformly according to the cycle, and adherence was recorded. During each follow-up, the medication adherence and adverse reactions were actively inquired about. All subjects received sleep hygiene guidance, weight management advice, and appropriate dietary management to minimize the interference of lifestyle factors on the study outcomes.

### 2.3. Observation indicators

This study sets up multi-dimensional observation indicators, covering general data, physiological parameters, laboratory tests, and sleep monitoring data, to systematically assess the antihypertensive effect, OSA improvement, and drug safety of spironolactone treatment.

#### 2.3.1. General data

Demographic and baseline clinical information were collected at the time of subject enrollment, including: age, sex, body mass index (BMI, kg/m^2^), medical history, and antihypertensive medication use.

#### 2.3.2. Blood pressure indicators

1. Office blood pressure (office BP): The office blood pressure was measured using a calibrated electronic sphygmomanometer by trained medical staff in the morning at a resting state, with 3 measurements taken to obtain an average value. Measurements were taken on day 0 (baseline), week 4, and week 12: systolic blood pressure (OSBP); diastolic blood pressure (ODBP).
2. 24-hour ambulatory blood pressure monitoring: A certified ambulatory blood pressure monitoring device was used for continuous 24-hour recording. Monitoring parameters included: 24-hour average systolic and diastolic blood pressure (24h ASBP, 24h ADBP); daytime average systolic and diastolic blood pressure (D SBP, D DBP, measured from 6:00 am to 10:00 pm); nighttime average systolic and diastolic blood pressure (N SBP, N DBP, measured from 10:00 pm to 6:00 am). Monitoring was performed on day 0 and week 12.

#### 2.3.3. Sleep apnea monitoring indicators

Portable sleep monitors were used to record respiratory events and blood oxygen changes from 10:00 pm to 6:00 am the following day. The following 2 indicators were analyzed: AHI: calculated as the total number of apneas and hypopneas per hour of sleep, assessing the severity of OSA; lowest arterial oxygen saturation (SaO_2_): reflecting the degree of nocturnal hypoxia. These 2 indicators were measured at baseline and at 12 weeks to compare differences between groups and changes within groups.

#### 2.3.4. Laboratory biochemical indicators

Key focus was placed on electrolyte and renal function indicators, specifically: blood potassium (K^+^), blood sodium (Na^+^): measured by ion-selective electrode method; blood urea nitrogen (BUN): measured by urease-glutamate dehydrogenase method; serum creatinine (Scr): measured by creatine oxidase method. Blood samples were collected on day 0, week 4, and week 12.

#### 2.3.5. Safety evaluation indicators

Adverse drug reactions (gastrointestinal discomfort, gynecomastia, muscle weakness); laboratory abnormalities (blood potassium ≥ 5.5 mmol/L, Na^+^ < 135 mmol/L, Scr increase > 30%, etc); whether premature discontinuation of medication or withdrawal due to adverse events occurred.

### 2.4. Adverse reactions and termination criteria

#### 2.4.1. Adverse reactions observation

Throughout the intervention period (12 weeks), all subjects were systematically monitored for adverse reactions, including clinical symptom assessments and laboratory tests.

Common adverse reactions monitoring: Digestive system: nausea, vomiting, bloating, decreased appetite, etc; Lactation and endocrine system: gynecomastia in males, menstrual disorders in females; Nervous system: dizziness, fatigue; Skin: rash, itching; and Others: weight changes, muscle soreness, etc.

Laboratory monitoring: Electrolyte abnormalities: blood potassium ≥ 5.5 mmol/L (hyperkalemia), blood sodium < 135 mmol/L (hyponatremia); renal function abnormalities: serum creatinine increased >30% or absolute value exceeded the upper limit; and significantly elevated BUN. These adverse events were recorded by the study physician, who determined their severity and made appropriate management decisions. The research team established an adverse reaction monitoring and emergency handling mechanism, with intervention suspension or termination if necessary.

#### 2.4.2. Trial termination or exclusion criteria

In the course of the study, the trial should be terminated or the patient excluded from the analysis cohort if any of the following occurs: The subject has poor compliance, unable to take medications according to the prescribed regimen or attend scheduled follow-ups; The subject alters the type or dose of their original antihypertensive medication; Severe adverse reactions occur, including but not limited to: persistent blood potassium ≥ 5.5 mmol/L or persistent blood sodium < 130 mmol/L; serum creatinine increases >1.5 times the normal upper limit or absolute value >133 μmol/L; gynecomastia in males or severe menstrual disorders significantly affecting quality of life; The patient voluntarily requests to withdraw from the study or is lost to follow-up; and During the study, severe underlying diseases or conditions that meet any of the exclusion criteria are discovered. Data from withdrawn patients will be retained, with reasons for withdrawal explained, and “intention-to-treat” (ITT) analysis and “safety analysis set” will be used in the data analysis.

### 2.5. Statistical analysis

Data entry and statistical analysis were conducted using SPSS version 29.0 (IBM Corp., Armonk). All statistical tests were two-sided, with a significance level set at *P* < .05. Categorical variables were summarized as frequencies (percentages) and compared using the chi-square test or Fisher exact test, as appropriate. Continuous variables were assessed for normality using the Shapiro–Wilk test. Normally distributed variables were presented as mean ± standard deviation and compared between groups using independent-sample *t*-tests; non-normally distributed variables were expressed as median [interquartile range] and analyzed with the Mann–Whitney *U* test. To compare post-intervention outcomes while adjusting for baseline differences, analysis of covariance (ANCOVA) was employed where applicable. The ANCOVA models included the post-treatment value as the dependent variable, treatment group as the fixed factor, and the corresponding baseline value as the covariate. Missing data were addressed using multiple imputation by chained equations within the intention-to-treat (ITT) population (n = 96). Variables included in the imputation model were: office blood pressure (OSBP 3% missing, ODBP 4% missing), 24-hour ambulatory blood pressure (24hASBP 5%, 24hADBP 6%, D SBP 5%, D DBP 5%, N SBP 6%, N DBP 7%), sleep parameters (AHI 8%, SaO_2_ 9%), and laboratory measures (K^+^ 4%, Na^+^ 3%, BUN 5%, Scr 4%). Five imputed datasets were generated, following SPSS default recommendations to ensure estimation stability while maintaining computational efficiency, and pooled estimates were calculated according to Rubin rules. In addition, a complete case analysis including only patients with no missing values was performed as a sensitivity check, which produced results consistent with the imputed datasets, supporting the robustness of the findings.

## 3. Results

### 3.1. Baseline data comparison

A total of 48 patients who completed the 12-week follow-up were included in the final per-protocol outcome analysis, with 24 patients in the spironolactone group and 24 patients in the control group. Among these, 38 were male and 10 were female. Baseline characteristics, including age, sex, BMI, office and 24-hour ambulatory blood pressure, AHI, SaO_2_, K^+^, Na^+^, BUN, Scr, number of comorbidities, and types of antihypertensive medications, were comparable between the 2 groups (*P* > .05), as shown in Table 1. No cases of gynecomastia were observed among male participants at baseline.

**Table 1:** Comparison of baseline characteristics in patients with mild obstructive sleep apnea (OSA) and refractory hypertension.

Indicators	Group A1 $\bar{x}\pm \;s$	Group A2 $\bar{x}\pm \;s$	*χ*^2^/*t*	*P*
n (cases)	24	24		
Stroke, n (%)	1 (4.2)	1 (4.2)	0.000*	1.000
Coronary heart disease, n (%)	4 (16.7)	3 (12.5)	0.167*	.683
Diabetes, n (%)	3 (12.5)	4 (20.8)	0.600*	.439
Dyslipidemia, n (%)	5 (20.8)	6 (25.0)	0.118*	.731
Types of antihypertensive drugs	3.12 ± 0.338	3.17 ± 0.381	−0.401†	.690
Number of male cases, n (%)	18 (75.0)	19 (79.2)	0.118*	.731
Age (yr)	54.46 ± 11.09	54.75 ± 15.18	−0.376†	.940
BMI (kg/m^2^)	26.221 ± 2.409	27.080 ± 1.948	−1.359†	.181
OSBP (mm Hg)	150.04 ± 6.104	148.58 ± 5.380	0.878†	.384
ODBP (mm Hg)	86.54 ± 5.875	87.08 ± 6.100	−0.313†	.755
24hASBP (mm Hg)	141.46 ± 3.189	139.88 ± 4.416	1.424†	.161
24hADBP (mm Hg)	78.67 ± 4.341	78.29 ± 4.418	0.297†	.768
D SBP (mm Hg)	144.33 ± 3.332	142.96 ± 4.309	1.237†	.222
D DBP (mm Hg)	80.58 ± 4.393	80.29 ± 4.268	0.233†	.817
N SBP (mm Hg)	138.38 ± 3.228	136.67 ± 4.613	1.487†	.144
N DBP (mm Hg)	77.67 ± 5.370	76.54 ± 4.452	0.790†	.434
K (mmol/L)	3.733 ± 0.337	3.608 ± 0.265	1.428†	.160
Na (mmol/L)	141.17 ± 1.435	141.131 ± 1.329	1.104†	.917
BUN (mmol/L)	5.595 ± 0.836	5.528 ± 0.937	0.262†	.795
Scr (μmol/L)	63.96 ± 12.682	65.83 ± 13.751	−0.491†	.626
AHI (order)	10.554 ± 2.962	9.821 ± 2.675	0.900†	.373
SaO2 (%)	86.500 ± 5.564	86.667 ± 4.007	−0.119†	.906

24hADBP = 24-hour average diastolic blood pressure, 24hASBP = 24-hour average systolic blood pressure, BP = blood pressure, BUN = blood urea nitrogen, CI = confidence interval, D DBP = daytime diastolic blood pressure, D SBP = daytime systolic blood pressure, N DBP = night diastolic blood pressure, N SBP = night systolic blood pressure, ODBP = office diastolic blood pressure, OSA = obstructive sleep apnea, OSBP = office systolic blood pressure, SaO_2_ = saturation of arterial oxygen.

* Chi-square test.

† *t*-test.

### 3.2. Comparison of antihypertensive effects of spironolactone on mild OSA with resistant hypertension

After 12 weeks of intervention, the spironolactone group showed significant reductions in both office and ambulatory blood pressure measurements compared with the control group. Specifically, OSBP was significantly lower in the spironolactone group (142.17 mm Hg vs 147.92 mm Hg), with an adjusted mean difference of − 5.75 mm Hg (95% CI: −8.84 to −2.66). The effect size for systolic BP was moderate, with a Cohen d of 0.53, indicating a clinically meaningful reduction. Similarly, ODBP also decreased significantly (82.54 mm Hg vs 86.21 mm Hg), with a difference of −3.67 mm Hg (95% CI: −6.46 to −0.88), and a Cohen d of 0.49, reflecting a moderate but significant improvement in blood pressure control.

Similar improvements were observed in ambulatory blood pressure measurements. The 24-hour average systolic blood pressure (24hASBP) decreased by −4.00 mm Hg (95% CI: −6.25 to −1.75), with a Cohen d of 0.44, suggesting a moderate clinical effect on systolic blood pressure control over 24 hours. The 24-hour average diastolic blood pressure (24hADBP) decreased by −3.54 mm Hg (95% CI: −5.48 to −1.60), indicating a moderate reduction in diastolic blood pressure, with a Cohen d of 0.47. Daytime systolic blood pressure (D SBP) was also reduced by −3.08 mm Hg (95% CI: −5.35 to −0.81), with a moderate effect size (Cohen d = 0.46), showing improved blood pressure control during waking hours. See Table 2.

**Table 2:** Comparison of outcome blood pressure in patients with mild obstructive sleep apnea (OSA) and refractory hypertension treated with spironolactone.

	Group			
Outcome blood pressure	Indicator in group A1 $\bar{x}\pm \;s$	Indicator in group A2 $\bar{x}\pm \;s$	*t*	*P*
n (cases)	24	24		
OSBP (mm Hg)	142.17 ± 6.532	147.92 ± 4.149	−3.640	<.001
ODBP (mm Hg)	82.54 ± 5.200	86.21 ± 4.634	−2.579	.013
24hASBP (mm Hg)	134.79 ± 3.349	138.79 ± 4.530	−3.479	.001
24hADBP (mm Hg)	74.13 ± 3.314	77.67 ± 3.535	−3.581	<.001
D SBP (mm Hg)	139.50 ± 3.912	142.58 ± 4.127	−2.656	.011
D DBP (mm Hg)	76.50 ± 4.273	79.96 ± 4.059	−2.875	.006
N SBP (mm Hg)	131.58 ± 3.229	136.42 ± 4.548	−4.245	<.001
N DBP (mm Hg)	73.33 ± 5.096	76.38 ± 4.241	−2.248	.029

24hADBP = 24-hour average diastolic blood pressure, 24hASBP = 24-hour average systolic blood pressure BP = blood pressure, BUN = blood urea nitrogen, CI = confidence interval, D DBP = daytime diastolic blood pressure, D SBP = daytime systolic blood pressure, N DBP = night diastolic blood pressure, N SBP = night systolic blood pressure, ODBP = office diastolic blood pressure, OSA = obstructive sleep apnea, OSBP = office systolic blood pressure, SaO_2_ = saturation of arterial oxygen.

These results indicate that spironolactone significantly improves both clinic and ambulatory blood pressure parameters in patients with mild OSA and resistant hypertension. The improvements in both clinic and ambulatory BP suggest that spironolactone may help reduce the cardiovascular risk associated with poorly controlled hypertension in this population.

**Figure 2.**
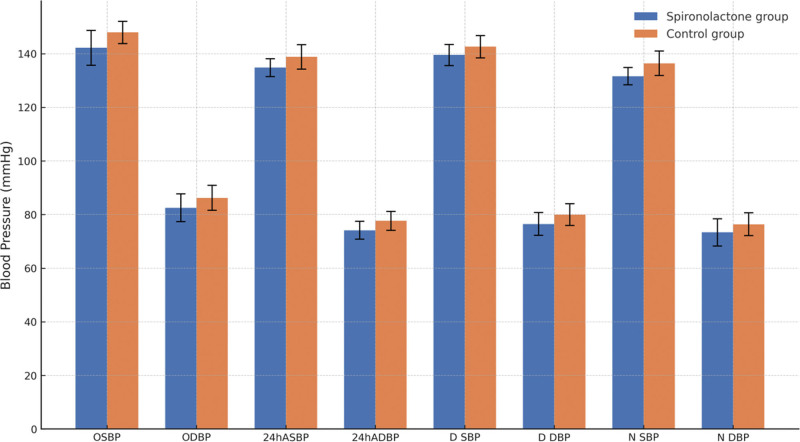
Effect of spironolactone on various blood pressure indicators in patients with mild obstructive sleep apnea and refractory hypertension.

The Figure 2 shows the comparison of OSBP, ODBP, 24hASBP, 24hADBP, D SBP, daytime diastolic blood pressure (D DBP), night systolic blood pressure (N SBP), and night diastolic blood pressure (N DBP) between the spironolactone and control groups.

### 3.3. Comparison of the effect of spironolactone on AHI in mild OSA with resistant hypertension

Compared to the control group, patients in the spironolactone group showed a significant reduction in the AHI after 12 weeks of treatment. The mean AHI in the spironolactone group was 7.55 events/hour, compared to 9.40 events/hour in the control group, with an adjusted mean difference of −1.85 events/hour (95% CI: −3.23 to −0.47). The effect size for AHI improvement was moderate, with a Cohen d of 0.44, indicating a clinically meaningful reduction in sleep-disordered breathing severity. These results suggest that spironolactone has a mild but statistically significant effect on improving the severity of sleep-disordered breathing in patients with mild OSA and resistant hypertension. See Table 3.

**Table 3:** Effect of spironolactone on apnea-hypopnea index (AHI) outcomes in patients with mild obstructive sleep apnea (OSA) and refractory hypertension.

	Group			
Outcome measures	Indicator in Group A1 $\bar{x}\pm \;s$	Indicator in Group A2 $\bar{x}\pm \;s$	*t*	*P*
n (cases)	24	24		
AHI (order/h)	7.558 ± 2.388	9.404 ± 2.515	−2.607	.012

AHI = apnea–hypopnea index.

### 3.4. Comparison of the effect of spironolactone on SaO_2_ in mild OSA with resistant hypertension

Patients receiving spironolactone also showed an improvement in oxygenation during sleep. The mean minimum SaO_2_ was higher in the spironolactone group compared to the control group (90.83% vs 87.50%), with an adjusted mean difference of +3.33% (95% CI: 0.93–5.73). This indicates that spironolactone treatment may reduce the degree of nocturnal hypoxemia in patients with mild OSA. See Table 4.

**Table 4:** Effect of spironolactone on saturation of arterial oxygen (SaO_2_) outcomes in patients with mild obstructive sleep apnea (OSA) and refractory hypertension.

	Group			
Outcome measures	Indicator in group A1 $\bar{x}\pm \;s$	Indicator in group A2 $\bar{x}\pm \;s$	*t*	*P*
n (cases)	24	24		
SaO2 (%)	90.833 ± 4.678	87.500 ± 3.765	2.719	.009

SaO_2_ = lowest arterial oxygen saturation.

### 3.5. Safety and tolerability outcomes

No cases of hyperkalemia (K^+^ ≥ 5.5 mmol/L) were reported in the spironolactone group. Serum potassium slightly increased from 3.73 ± 0.34 mmol/L to 4.15 ± 0.29 mmol/L (*P* < .05), while creatinine and BUN levels remained stable (*P* > .05). No participants experienced gynecomastia, menstrual disturbances, or gastrointestinal adverse events. No patients withdrew due to intolerance. Medication adherence remained above 95% throughout the study.

## 4. Discussion

In this retrospective cohort study, we evaluated the clinical effectiveness and safety of spironolactone as an adjunct therapy in patients with mild OSA and resistant hypertension. Compared with standard antihypertensive therapy alone, spironolactone use was associated with greater reductions in office and ambulatory blood pressure, a modest improvement in AHI, and increased minimum oxygen saturation. These results suggest that spironolactone may provide dual benefits in this patient population. The results showed that after 12 weeks of intervention with 20 mg/day of spironolactone combined with the original antihypertensive treatment regimen, the experimental group had significant reductions in office systolic and diastolic blood pressures, and 24-hour ambulatory blood pressure, as well as daytime and nighttime average blood pressures were all significantly better than those of the control group, with statistically significant differences (*P* < .05); AHI significantly decreased, and minimum SaO_2_ significantly increased, suggesting that spironolactone not only has good efficacy in blood pressure control but also has a certain degree of intervention effect on sleep-disordered breathing. In terms of safety, no hyperkalemia or significant renal dysfunction occurred in the experimental group during the intervention period, and no patients discontinued treatment due to adverse reactions, indicating good overall tolerability. It is worth noting that existing literature mainly focuses on the effects of aldosterone antagonism in moderate to severe OSA or primary aldosteronism patients, while mild OSA patients, due to their subtle symptoms and poor compliance, are often overlooked. However, this population already exhibits changes in the internal environment and a tendency for autonomic nervous dysfunction before cardiovascular risks fully manifest, which presents important intervention value. This study, by selecting a population with mild OSA combined with multi-drug-resistant hypertension, applied spironolactone without CPAP treatment, effectively avoided the interference of low CPAP compliance and focused on a more universal and practical drug intervention approach, making it highly applicable in real-world settings. According to the “2018 Revised Chinese Guidelines for the Prevention and Treatment of Hypertension”^[9]^ and the “2018 Guidelines for Primary Care Management of Adult Obstructive Sleep Apnea,”^[10]^ resistant hypertension patients should consider the evaluation and intervention of potential secondary factors such as OSA. However, in mild OSA patients, there is still a lack of standardized treatment recommendations, and evidence supporting CPAP treatment inthis group is limited. Therefore, exploring drug treatment strategies that have both antihypertensive and anti-OSA potential is particularly important. Spironolactone, by antagonizing aldosterone and reducing water and sodium retention, helps alleviate upper airway tissue edema and improve nighttime ventilation conditions, thus achieving a dual improvement in blood pressure and OSA severity. This mechanistic basis and clinical observation are reflected in this study.

As a classic aldosterone receptor antagonist, spironolactone lowers blood pressure by inhibiting aldosterone binding to mineralocorticoid receptors in the distal tubules and collecting ducts, thereby reducing sodium reabsorption and potassium excretion. This promotes natriuresis, reduces plasma volume and peripheral resistance, and is particularly effective for volume-dependent hypertension.^[11–14]^ In resistant hypertension, persistent RAAS overactivation leads to elevated aldosterone levels, causing fluid retention and sympathetic excitation, which further complicates blood pressure control.^[15–17]^ OSA patients often exhibit heightened RAAS activity, with serum aldosterone levels positively correlated with OSA severity. Aldosterone may exacerbate both hypertension and OSA through fluid overload and sympathetic stimulation. Elevated aldosterone contributes to upper airway mucosal edema, increased tongue volume, and narrowed pharyngeal space, thereby increasing airway collapsibility during sleep and worsening OSA severity.^[18–20]^ In this study, 12 weeks of spironolactone therapy not only improved blood pressure but also significantly reduced AHI and increased minimum SaO_2_. These effects may result from the reduction of upper airway tissue edema via modulation of fluid balance, indirectly improving nocturnal ventilation and reducing hypoxic events and arousals.^[21]^ Notably, patients with current CPAP usage were excluded from this study to isolate the pharmacologic effects of spironolactone. While CPAP remains the first-line treatment for moderate-to-severe OSA, its role in mild OSA is less definitive, particularly in patients with poor adherence or limited access. Previous studies have shown that CPAP may have modest antihypertensive effects in this subgroup, but adherence challenges often limit its effectiveness. Given the dual benefit of spironolactone in lowering blood pressure and improving sleep-disordered breathing in our study, future investigations should explore its comparative or adjunctive role alongside CPAP in broader OSA populations.

In this study, spironolactone demonstrated a favorable short-term safety profile in patients with OSA and resistant hypertension. During the 12-week follow-up period, no severe drug-related adverse events were observed, and no patients discontinued treatment due to intolerance. Specifically, no cases of gynecomastia, gastrointestinal discomfort, dizziness, or fatigue were reported. Laboratory monitoring showed stable renal function, with no clinically significant changes in serum creatinine or BUN. Although serum potassium levels increased modestly compared with baseline, all values remained within the normal physiological range (<5.5 mmol/L) and were not associated with clinical manifestations of electrolyte imbalance. This mild elevation is consistent with the known pharmacological effects of aldosterone receptor antagonism and may reflect correction of subclinical hypokalemia related to renin–angiotensin–aldosterone system overactivation and chronic intermittent hypoxia in patients with OSA.^[22,23]^ Previous studies have similarly demonstrated that spironolactone at doses of 20 to 50 mg/day is generally well tolerated in patients with resistant hypertension and preserved renal function, with a low incidence of clinically relevant hyperkalemia.^[24,25]^ Long-term observational data further support the safety of spironolactone, even with modest increases in serum potassium.^[26–30]^ Nevertheless, these findings should be interpreted cautiously. The study population was relatively young, with normal baseline renal function and low baseline serum potassium levels, which may limit the generalizability of the safety outcomes to older patients or those with chronic kidney disease. Careful patient selection and regular monitoring of serum electrolytes and renal function, particularly during treatment initiation, remain essential when prescribing spironolactone in clinical practice.

Recent evidence has highlighted the important role of mineralocorticoid receptor antagonists (MRAs) in the management of resistant hypertension. MRAs, including spironolactone, have been shown to be highly effective as add-on therapies for blood pressure control, even at relatively low doses, while maintaining an acceptable safety profile.^[31]^ Experimental studies suggest that MRAs may also mitigate cardiovascular dysfunction associated with intermittent hypoxia, providing mechanistic support for their potential benefits in patients with OSA.^[32]^ Clinical data indicate that spironolactone can reduce the severity of OSA, likely by decreasing upper airway edema and improving nocturnal ventilation.^[33,34]^ These findings align with the results of the present study, supporting the dual effect of spironolactone in lowering blood pressure and improving sleep-disordered breathing. Further large-scale, multicenter, and long-term studies are warranted to validate these findings in broader OSA populations.

This study has several limitations. First, its retrospective design introduces risks of selection and information bias, precluding firm causal inference. Second, the sample was relatively small and from a single center, which reduces external validity. Third, although clinical endpoints were objectively measured, residual confounding (e.g., diet, medication adherence, aldosterone status) cannot be excluded. Finally, the follow-up was short, so long-term effectiveness and safety remain uncertain. Larger, multicenter studies – preferably prospective and randomized with longer follow-up – are needed to confirm these findings and better define the role of spironolactone in patients with mild OSA and resistant hypertension.

In this study, spironolactone demonstrated potential benefits in reducing blood pressure and improving sleep-related parameters in patients with mild OSA and resistant hypertension. While these findings suggest that spironolactone could be a promising adjunctive treatment in this population, the small sample size and single-center design limit the generalizability of the results. Further, larger, and multicenter studies are required to confirm these findings and to establish stronger evidence for the role of spironolactone in managing mild OSA and resistant hypertension.

## Acknowledgments

Baoding Science and Technology Plan Project, Project No. 2341ZF145.

## Author contributions

**Conceptualization:** Chao Fang, Bo Ma.

**Data curation:** Chao Fang, Bo Ma.

**Formal analysis:** Chao Fang, Bo Ma.

**Funding acquisition:** Chao Fang.

**Investigation:** Nuan Xiao.

**Methodology:** Chao Fang, Bo Ma.

**Project administration:** Nuan Xiao, Hongmei Zhao, Simin Ren.

**Resources:** Nuan Xiao, Hongmei Zhao, Simin Ren.

**Software:** Nuan Xiao, Hongmei Zhao, Simin Ren.

**Supervision:** Nuan Xiao, Simin Ren.

**Validation:** Nuan Xiao, Simin Ren.

**Visualization:** Chao Fang, Bo Ma.

**Writing – original draft:** Chao Fang, Bo Ma, Nuan Xiao, Hongmei Zhao, Simin Ren.

**Writing – review & editing:** Chao Fang, Bo Ma, Nuan Xiao, Hongmei Zhao, Simin Ren.
